# Do restrictive gender attitudes and norms influence physical and mental health during very young Adolescence? Evidence from Bangladesh and Ethiopia

**DOI:** 10.1016/j.ssmph.2019.100480

**Published:** 2019-11-20

**Authors:** Sarah Baird, Zulfiqar A. Bhutta, Bassam Abu Hamad, Joan Hamory Hicks, Nicola Jones, Jennifer Muz

**Affiliations:** a Department of Global Health, Milken Institute School of Public Health, George Washington University, Washington, D.C, 20052, USA; b Centre for Global Child Health, The Hospital for Sick Children, 525 University Avenue, Suite 702, Toronto, Canada; c Canada and Centre of Excellence in Women and Child Health, The Aga Khan University, Karachi, Pakistan; d School of Public Health, Al Quds University, Abu Dees, POB 89, Jerusalem, Palestinian Authority; e Department of Economics, University of Oklahoma, 308 Cate Center Drive, Norman, OK, 73072, USA; f Overseas Development Institute, 203 Blackfriars Rd, London, UK

**Keywords:** Adolescent health, Mental health, Gender norms, LMICs

## Abstract

Adolescence is seen as a window of opportunity for intervention but also as a time during which restrictive gender attitudes and norms become more salient. This increasingly gendered world has the potential to profoundly influence adolescents’ capabilities, including their physical and mental health. Using quantitative data on 6,500 young adolescents (10–12) from the Gender and Adolescence: Global Evidence (GAGE) program, this paper analyses the association between restrictive gender attitudes (RGAs) at the individual level and restrictive gender norms (RGNs) at the community level and physical and mental health in Bangladesh and Ethiopia. We find significant associations between RGAs and RGNs and height-for-age z-scores, body mass index z-scores, self-reported health, adolescent hunger, psychological well-being, and self-esteem. We find no relationship between RGAs or RGNs and illness. We also find heterogeneity across country and urbanicity. We find surprisingly limited variation by gender, and the differences we do see point to important vulnerabilities for both boys and girls. Our results point to the powerful role that distal factors such as culture and beliefs, as manifested through RGAs and RGNs, can play in shaping health outcomes for both boys and girls and suggest important next steps for future research and policy.

## Highlights

• Restrictive gender attitudes (RGAs) and norms (RGNs) influence physical and mental health during adolescence.
• RGAs and RGNs impact the health of both boys and girls.
• RGAs and RGNs impact health differently in urban versus rural areas.
• Policies to improve health in adolescents must consider restrictive gender attitudes and norms.

## 1 Introduction

There is increasing recognition, on the part of both scientists and development actors, that adolescence is a key window of opportunity for intervention (Sheehan et al., 2017; Steinberg, 2015; UNFPA, 2014)—perhaps second only to the first 1,000 days of life. This is not only because of the physical changes associated with puberty but also due to shifts in the child's status within the family and community as s/he approaches maturity (GAGE Consortium, 2017). Although adolescence is a time for promoting an individual's development, it is also a time when social norms become increasingly influential in shaping what young people do and are expected to do (Chung & Rimal, 2016). In particular, restrictive gender norms begin to play a greater role in shaping adolescent trajectories during the second decade of life and into early adulthood (Basu, Zuo, Lou, Acharya, & Lundgren, 2016; John, Stoebenau, Ritter, & Edmeades, 2016; Kågesten et al., 2016; McCarthy, Brady, & Hallman, 2016; Mmari et al., 2018).

In high-income countries (HICs), the literature on the role of restrictive gender norms during adolescence focuses on risk-taking behaviors such as drinking alcohol and smoking (see, for example, Elek, Miller-Day, & Hecht, 2006). For adolescent girls in low- and middle-income countries (LMICs), the “endorsement of gender stereotypes” can be particularly salient (Chung & Rimal, 2016). The years of early adolescence, in particular, frequently narrow the world that girls inhabit, as they must leave behind their comparatively free childhoods to follow the prevailing gendered adult pathways, which typically emphasize female domestic and caregiving work responsibilities and control of female sexuality (Harper, Jones, Ghimire, Marcus, & Kyomuhendo Bantebya, 2018; Kabeer, 2003; Nussbaum, 2011).

For boys, by contrast, the world tends to open up during early adolescence, but this is not without its costs. Adolescent boys are more likely to engage in and experience physical violence (Betancourt, Agnew-Blais, Gilman, Williams, & Ellis, 2010; Buller, 2015), to die from unintentional injuries, to engage in substance use, and to commit suicide (Blum, Mmari, & Moreau, 2017; Levtov, Barker, Contreras-Urbina, Heilman, & Verma, 2014). As Mmari et al. (2018) note, “These differences in perceptions of vulnerability and related mobility are markers of a gender system that separates young women and men's roles, responsibilities and behaviours in ways that widen gender power imbalance with lifelong social and health consequences for people of both sexes.” Jones, Tefera, Presler-Marshall, and et al (2015) further argue that individual-level empowerment is not enough for sustained change to take root; rather “it will necessitate broader inter-sectoral and tailored attention to the web of gender norms binding them [adolescent girls] to the past” (539).

Restrictive gender attitudes and norms—hereafter RGAs and RGNs—have the potential to profoundly influence adolescents’ capabilities, including their physical and mental health. However, we do not yet have a full understanding of the extent to which RGAs and RGNs influence the health of very young adolescents (10–12) in LMICs and how this may differ by gender and context. Using new quantitative data from the Gender and Adolescence: Global Evidence (GAGE) program (discussed in Section 2) on close to 6,500 adolescent boys and girls in Bangladesh and Ethiopia, this study attempts to add to the limited literature in this space.

To understand the potential for RGAs and RGNs to influence health in LMICs, it is useful to provide a brief framework. Cislaghi and Heise (2018) present an adaptation of the ecological framework in which individual (e.g., attitudes), material (e.g., money), social (e.g., social support), and institutional (e.g., laws) factors overlap to influence people's actions. At the intersection of these factors is where gender, power, and social norms operate (Cislaghi & Heise, 2018). The centrality of RGNs in this framework highlights their ability, alongside RGAs, to influence health.

In terms of *physical health*, adolescence is a time of rapid physiological, sexual, neurological, and behavioral changes, and adequate nutrition is essential for achieving full growth potential (Das et al., 2017). RGNs often imply that girls face unfair food distribution in the household (Oniang'o & Mukudi, 2002) or face an excessive burden of physically demanding chores (e.g., fetching water) (Blackden & Wodon, 2006; Dickert & Dodson, 2004). This, combined with the fact that adolescents have greater nutritional needs than adults (Das et al., 2017), leads adolescent girls in LMICs to have poorer nutritional profiles than their counterparts in HICs (Caleyachetty et al., 2018; Keats et al., 2018; Lassi, Moin, Das, Salam, & Bhutta, 2017). Malnutrition during adolescence is also highly contextual (Caleyachetty et al., 2018) and frequently related to the state of maternal nutrition (Di Cesare et al., 2014)—which itself is influenced by RGNs.

Adolescence is also a time of heightened *psychosocial vulnerability*, with half of all mental illnesses beginning by age 14 years and neuropsychiatric disorders now being the leading cause of disability in adolescence (Patel, Flisher, Hetrick, & McGorry, 2007; WHO, 2014). RGNs play a substantial role in girls' greater susceptibility to mental health problems. Even in childhood, they are less likely than boys to have the unstructured time to play and to “be” that is critical to the development of identity, self-esteem, and broader well-being (Jones et al., 2017g refugee girls in Ethiopia, Stark et al. (2018) found that although girls’ attitudes towards gender-inequitable norms were not predictive of low social esteem, collective peer norms around the same gender-inequitable statements were. The parents of adolescent girls tend to see their daughters as more compliant and useful, so give them a disproportionate share of chores, which makes girls more likely to be socially isolated and less likely to have opportunities to pursue their own interests (John et al., 2016; Samuels, Jones, & Abu Hamad, 2017). Adolescence all too often deepens this isolation, particularly in societies in which girls see their mobility restricted and where they marry as children and thus lose the daily support of their natal families (Abu Hamad et al., 2018; Edmeades, Hayes, & Gaynair, 2014; Jones et al., 2015, Jones et al., 2015; WHO, 2014).

This paper builds on existing literature and utilizes the framework proposed by Cislaghi and Heise (2018) to answer the following three research questions using GAGE data: 1) are RGAs and RGNs associated with worse physical and mental health outcomes among adolescents in Bangladesh and Ethiopia? 2) Is this association stronger for attitudes or norms? 3) How do these impacts vary across a number of key dimensions, including gender and location (country of residence and urban or rural setting)?

## 2 Methods

### 2.1 GAGE study design

This paper draws on baseline findings from the GAGE program, which is longitudinally following 18,000 adolescents and their caregivers across six LMICs over a nine-year period (2015–2024). The aim of the program is to use mixed quantitative and qualitative research methods to discover what works to support the development of adolescent girls' and boys’ capabilities during the second decade of life (age 10–19 years).

**Fig. 1:**
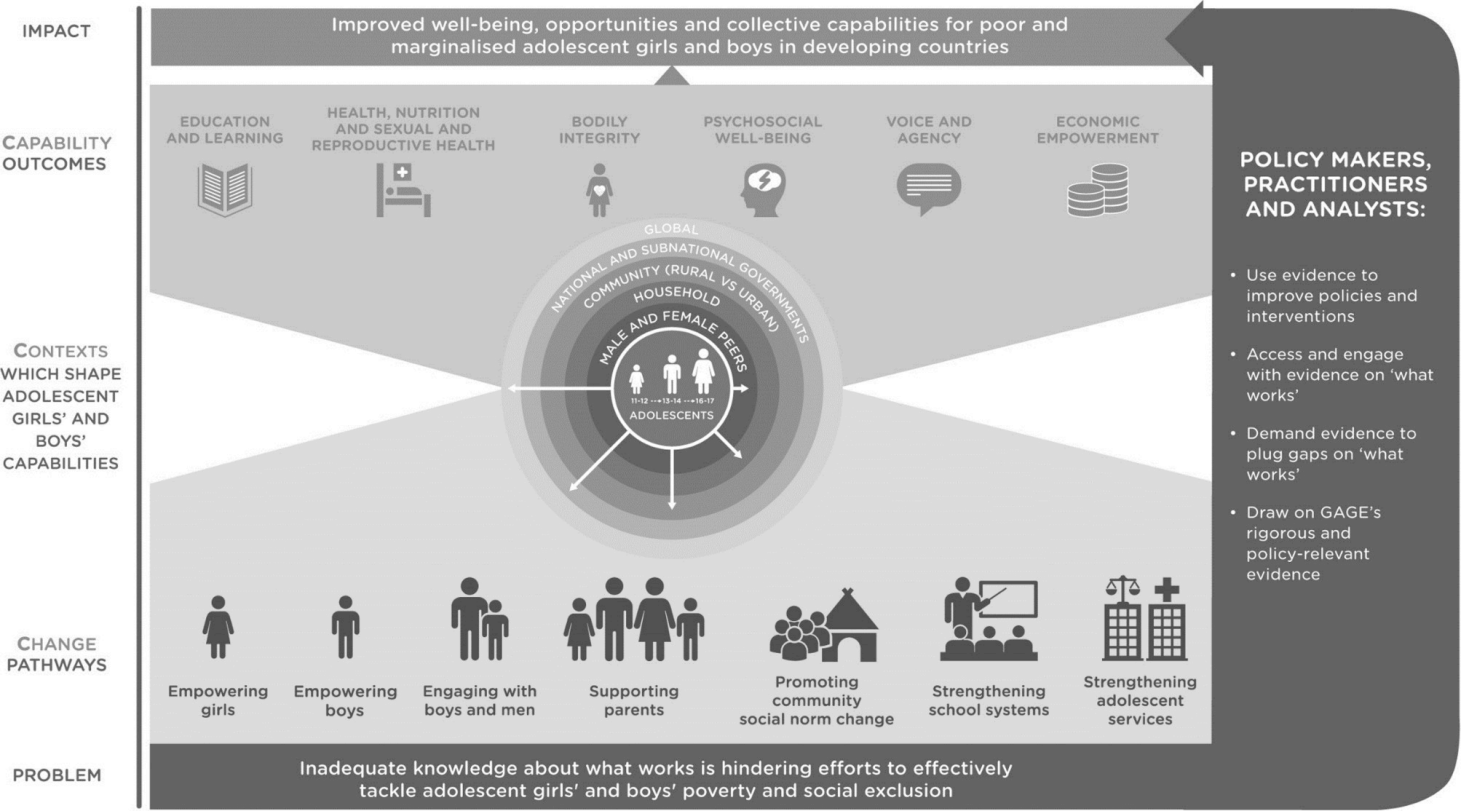
Gender and Adolescence: Global Evidence (GAGE) conceptual framework. Source: GAGE Consortium, 2019 in press.

Underpinning GAGE is a conceptual framework focusing on the interconnectedness of adolescent capabilities, the diverse contexts in which adolescents in LMICs live, and programmatic change strategies operating at micro, meso, and macro levels (see Fig. 1). Like the framework presented by Cislaghi and Heise (2018), the GAGE conceptual framework is based on an ecological model that places the adolescent girl at the center of a series of influences ranging from the individual to household and global levels. It is then both the adolescent's own attitudes and the surrounding social norms, as well as other household, community, and institutional structures, that influence capability outcomes including physical and mental health (GAGE Consortium, 2019 in press).

This paper draws on data collected during 2017 and 2018 from the two GAGE countries where baseline data collection is complete: Bangladesh and Ethiopia. The study took place in three locations in Bangladesh (Dhaka [urban], Chittagong [rural and urban] and Sylhet [rural and urban]) and six locations in Ethiopia (Debre Tabor [urban] and South Gondar [rural] in Amhara regional state, Ziway [urban] and East Hararghe [rural] in Oromia regional state, Dire Dawa City Administration [urban], and Zone 5 [rural] in Afar regional state), with slight variations in the sampling strategy across locations. The focus of this paper is on 10- to 12-year-old adolescents, who were surveyed in all locations (except Ziway). The older cohort (15–17-year-old adolescents) was only surveyed in a subset of urban areas.

### 2.2 Data collection procedures

In both Dhaka and Ethiopia, a household census was used to identify the population of potential respondents. In Chittagong and Sylhet, a school-based census was used. In addition to the random sample, in all locations, GAGE sought to purposely sample out-of-school adolescents and adolescents with disabilities. This process resulted in 1,623 surveys in Bangladesh and 4,866 surveys in Ethiopia. Sampling weights, reflecting the probability of selection into the study sample, are used to make the results representative of the target population in the study area. For more details on the sampling strategy, refer to Jones, Baird, and Lunin (2018).

Written or verbal consent, depending on literacy, was obtained for caregivers and married adolescents, with written or verbal assent obtained for all unmarried adolescents younger than 18 years. Surveys were translated into local languages (Bangla in Bangladesh and either Afaan Oromo or Amharic in Ethiopia) and were tested extensively. Data collection took place through face-to-face interviews with enumerators. In most cases, the enumerator was from the same region and was the same sex as the adolescent. Enumerators were trained extensively in the wording of the questions and how to appropriately interact with adolescents.

### 2.3 Measures

#### 2.3.1 Gender attitudes and norms

Our main independent variables of interest are measures of restrictive gender attitudes and social norms. We developed a set of context- and age-specific gender attitude and social norms questions drawing on a variety of established sources in attitude and norm measurement (Baird, McIntosh, & Özler, 2017; El Feki et al., 2017; Global Early Adolescent Study, 2018; Levtov et al., 2018; Lundgren, Beckman, Chaurasiya, Subhedi, & Kerner, 2013; Nanda, 2011; Singh, Verma, & Barker, 2013; Vu et al., 2017; Waszak, Severy, Kafafi, & Badawi, 2000). We focus on a set of domains that we deemed to be particularly relevant during adolescence: education, time use, financial inclusion and economic empowerment, relationships and marriage, and sexual and reproductive health. Our starting point for the development of these domains was Sen's ideas of capabilities as basic human functioning to lead a life the individual deems worth living—so more than human capital—with an emphasis on ability to exercise voice and agency (Sen, 1984; Sen, 2004). Then we looked at Nussbaum's more elaborate set of capabilities, which were framed through a feminist lens (Nussbaum, 2011). Nussbaum (2011) identifies 10 core capabilities that all democracies should support, and we clustered these capabilities according to priorities the literature highlights, vis-à-vis developmental milestones and imperatives during the second decade of life. Finally, we wanted to ensure our clustering resonated with the rights-based framing of the United Nations Convention on the Rights of the Child and focus on rights to survival, development, protection, and participation.

The set of attitude questions measure what the respondent thinks—for example, whether s/he thinks “girls and boys should share household tasks equally.” The set of norms questions captures descriptive norms (what the respondent thinks others do) and injunctive norms (what the respondent believes others think that s/he should do) (Kallgren, Reno, and Cialdini, 2000; Reid, Cialdini, and Aiken, 2010; Stefanik & Hwang, 2017). For example, “adults in my community expect adolescent girls to get married before the age of 18.”

Given that attitude and norm questions can be challenging for respondents, the questions were carefully translated and back-translated, as well as piloted, to ensure they resonated with the study population. We also focused on attitudes and norms that resonated with adolescents during formative qualitative work in diverse parts of each country (rural, urban, pastoralist, and in Ethiopia, different religious and ethnic communities) and comprised issues that were common across the GAGE countries. Furthermore, enumerators were trained extensively on question administration, in particular on the importance of privacy in the interview, and conveying with both words and body language that there are no right or wrong answers to the questions. During training, time was allocated for ensuring that the enumerators understood the intent of the of the attitudes and norms statements. In tandem with this, translations were discussed in detail and refined as needed.

We utilize a total of 16 attitudes and 14 norms questions. Appendix Table A.1, Table A.2 summarize the full list of gender attitude measures (at the individual level) for Bangladesh and Ethiopia, with Appendix Table A.3, Table A.4 summarizing the norm measures (at the community level). Appendix Table A5 provides additional information on the primary source of each measure. It is important to note that measures of attitudes and norms likely suffer from some social desirability bias (Dhar, Jain, & Jayachandran, 2018), so should be interpreted with this in mind.

For attitudes and social norms questions, we use the same coding structure. For each statement, respondents are assigned a “1” if they agreed or partially agreed and a “0” if they disagreed in cases where agreement suggested a gendered response, and the reverse if agreement suggested a nongendered response. The final measure is created by summing the values of the 16 attitudes questions and 14 norms, with larger values reflecting highly gender-inequitable attitudes and norms. We then create standardized measures (mean 0, SD 1) of the attitude and norm measures for analysis. This is done at the individual level for attitudes and the community level for norms. Summary statistics for the summed measure are presented in Table 1a (Bangladesh) and Table 1b (Ethiopia). In Ethiopia, the community norms had a Cronbach's alpha of 0.85 and the individual attitudes had a Cronbach's alpha of 0.64, while in Bangladesh the community norms had a Cronbach's alpha of 0.84 and the individual attitudes had a Cronbach's alpha of 0.45. Although the norms have high reliability, the Cronbach's alpha for attitudes is adequate to low (Tavakol and Dennick, 2011; Taber, 2017), which should be kept in mind when interpreting the results.

**Table 1a:** Descriptive statistics of attitudes, norms, physical health, and mental health (Bangladesh).

	Overall	Male	Female	Rural	Urban
	Mean (SD)				
Attitudes and Normsrowhead					
Attitudes (0–16; higher scores are more gendered)	5.619	5.349	5.897	5.954	5.521
	(2.180)	(2.028)	(2.294)	(2.022)	(2.215)
Norms (0–14; higher scores are more gendered)	7.343			7.401	7.326
	(1.295)			(1.082)	(1.351)
Physical Healthrowhead					
Height for age z-score	0.331	0.190	0.476	0.130	0.391
	(1.293)	(1.184)	(1.383)	(1.395)	(1.256)
Body mass index for age z-Score	-0.055	-0.035	-0.075	-0.450	0.062
	(1.463)	(1.398)	(1.527)	(1.298)	(1.488)
= 1 If self-reported health good or very good	0.890	0.896	0.884	0.951	0.873
	(0.312)	(0.305)	(0.320)	(0.215)	(0.334)
= 1 If adolescent experienced health symptom in past 4 weeks or serious illness or injury in past 12 months	0.170	0.113	0.228	0.145	0.177
	(0.375)	(0.317)	(0.420)	(0.353)	(0.382)
= 1 If adolescent experienced hunger in the past four weeks	0.0351	0.0303	0.0400	0.0618	0.0272
	(0.184)	(0.171)	(0.196)	(0.241)	(0.163)
Mental Healthrowhead					
General Health Questionnaire 12 Index (0–12; higher indicates worse mental health)	1.228	1.227	1.229	0.707	1.382
	(1.461)	(1.438)	(1.486)	(1.204)	(1.495)
Rosenberg's Self Esteem Scale (10–40; larger values equal higher self-esteem)	30.24	30.22	30.25	30.67	30.11
	(3.498)	(3.515)	(3.482)	(3.348)	(3.532)
Control Variablerowheads					
Asset deciles	6.570	6.402	6.744	4.669	7.129
	(2.859)	(2.877)	(2.831)	(2.968)	(2.572)
Indicator for having a friend he/she trusts	0.932	0.938	0.927	0.935	0.932
	(0.251)	(0.241)	(0.261)	(0.247)	(0.252)
Index of talking to primary female caregiver (0–4)	2.262	2.299	2.224	2.161	2.292
	(0.633)	(0.631)	(0.633)	(0.652)	(0.624)
Indicator for female	0.507	1.000	0,000	0.496	0.510
	(0.500)	(0.000)	(0.000)	(0.501)	(0.500)
Indicator for urban	0.773	0.778	0.768	0.000	1.000
	(0.419)	(0.416)	(0.423)	(0.000)	(0.000)
Sample size: Individuals	1623	841	782	451	1172
Sample size: Communities	111	111	111	36	75

*Notes:* This table summarizes the independent and dependent variables from the Gender and Adolescence: Global Evidence (GAGE) Bangladesh quantitative survey. Means are weighted to make them representative of the study communities. We restrict our sample to adolescents those that have defined attitude and norm measures. There are small differences in sample sizes across outcomes. For ease of presentation, the sample size at the bottom of each column reflects the maximum sample size for that subsample; the specific sample size for each outcome can be seen in the regression tables.

**Table 1b:** Descriptive statistics of attitudes, norms, physical health, mental health, and controls (Ethiopia).

	Overall	Male	Female	Rural	Urban
	Mean (SD)				
Attitudes and Norms					
Attitudes (0–16, higher scores are more gendered)	6.591	6.490	6.683	6.708	5.562
	(2.776)	(2.800)	(2.751)	(2.771)	(2.599)
Norms (0–14, higher scores are more gendered)	9.465			9.663	7.733
	(1.381)			(1.253)	(1.243)
Physical Health					
Height for age z-score	-0.780	-0.907	-0.666	-0.817	-0.458
	(1.344)	(1.326)	(1.349)	(1.348)	(1.260)
Body mass index for age z-score	-1.151	-1.137	-1.163	-1.213	-0.607
	(0.974)	(0.993)	(0.957)	(0.927)	(1.186)
= 1 If self-reported health good or very good	0.882	0.881	0.883	0.879	0.907
	(0.323)	(0.324)	(0.322)	(0.326)	(0.291)
= 1 If adolescent experienced health symptom in past 4 weeks or serious illness or injury in past 12 months	0.160	0.158	0.161	0.151	0.231
	(0.366)	(0.365)	(0.368)	(0.358)	(0.422)
= 1 If adolescent experienced hunger in the past four weeks	0.202	0.169	0.232	0.209	0.143
	(0.402)	(0.375)	(0.422)	(0.407)	(0.351)
Mental Health					
General Health Questionnaire 12 Index (0–12, higher indicates worse mental health)	0.959	0.924	0.991	0.946	1.074
	(1.591)	(1.614)	(1.570)	(1.589)	(1.605)
Rosenberg's Self Esteem Scale (10–40; larger values equal higher self-esteem)	29.18	29.14	29.22	29.11	29.84
	(3.155)	(3.146)	(3.164)	(3.123)	(3.359)
Control Variables					
Asset deciles	5.351	5.363	5.341	5.373	5.161
	(2.635)	(2.634)	(2.637)	(2.495)	(3.639)
Indicator for having a friend he/she trusts	0.762	0.790	0.737	0.765	0.734
	(0.426)	(0.408)	(0.440)	(0.424)	(0.442)
Index of talking to primary female caregiver (0–4)	2.089	2.078	2.099	2.026	2.638
	(1.148)	(1.121)	(1.172)	(1.148)	(0.992)
Indicator for female	0.474	1.000	0.000	0.472	0.493
	(0.499)	(0.000)	(0.000)	(0.499)	(0.500)
Indicator for urban	0.102	0.106	0.0985	0.000	1.000
	(0.303)	(0.308)	(0.298)	(0.000)	(0.000)
Sample size: Individuals	4866	2712	2154	4126	740
Sample size: Communities	218	218	218	175	43

Notes: This table summarizes the independent and dependent variables from the Gender and Adolescence: Global Evidence (GAGE) Ethiopia quantitative survey. Means are weighted to make them representative of the study communities. We restrict our sample to those that have defined attitude and norm measures. There are small differences in sample sizes across outcomes. For ease of presentation, the sample size at the bottom of each column reflects the maximum sample size for that subsample; the specific sample size for each outcome can be seen in the regression tables.

#### 2.3.2 Physical health

In this paper, we focus on five measures related to physical health. We construct age- and gender-adjusted height-for-age z-scores (HAZ) and body mass index z-scores (BMIZ). According to Campisi, Carducci, Söder, and Bhutta (2018), “long-term exposure to an inadequate diet, repeated exposure to infection and the inability to engage in ‘catch-up’ growth are characteristics of chronic undernutrition and the resulting impaired linear growth can be quantified in height-for-age z scores (HAZ) scores.” BMIZ, on the other hand, gives a sense of whether the adolescent's nutritional requirements are being met and are a result of long- or short-term deficiencies (Benny, Boyden, & Penny, 2018). The z-scores are constructed following Vidmar, Carlin, Hesketh, and Cole (2004), using data on height and weight collected from all adolescents. In both countries, two measures of height and weight were taken and we utilize the average in our construction of the variable.

Our measure of self-reported health asks the adolescent to answer the following: “In general, would you say your health is …” with response options of “very good,” “good,” “fair,” “poor,” and “very poor” (Health Information National Trends Survey (HINTS)-5, 2018). We create an indicator of good self-reported health that is equal to 1 if the response option of “very good” or “good” is chosen, and 0 otherwise. For experience of illness or injury, we create an indicator that takes on a value of 1 if the adolescent has experienced any of a series of health symptoms in the past four weeks or a serious illness or injury in the past 12 months.

For adolescent hunger, we asked “Now I am going to read you a statement that some children have made about their food situation. ‘I feel hungry, because there is not enough food to eat.’ In the last four weeks, this has happened many times, 1–2 times, or never?” We create a variable equal to 1, indicating that the adolescent is food insecure, if s/he answers, “many times” or “1–2 times.” Summary statistics of these variables can be found in Table 1b, Table 1aa and 1b

#### 2.3.3 Mental health

We analyse two measures of mental health: a version of the General Health Questionniare-12 (GHQ-12) developed by Goldberg and Blackwell (1970) and Goldberg and Williams (1988), and Rosenberg's self-esteem scale (RSES) (Rosenberg, 1965). The GHQ-12 was developed as a screening instrument to detect individuals who have the common mental health problems of anxiety, depression, and social withdrawal (Jackson, 2007). Each item is rated on a 4-point scale (less than usual, no more than usual, rather more than usual, or much more than usual). We utilize binary scoring (1-0-0-0 if positive statement and 0-0-1-1 if negative statement), with summed scores ranging from 0 to 12 and higher scores indicative of increased psychological distress. In our sample, the GHQ-12 has a Cronbach's alpha of 0.71 in Ethiopia and 0.55 in Bangladesh.

The RSES is a 10-item scale, originally designed to measure self-esteem of high school students, on a 4-point Likert scale (from “strongly agree” to “strongly disagree”) (Rosenberg, 1965). The final scale takes a value between 10 and 40, where a higher value indicates greater self-esteem. The RSES has been used globally and shown support for cross-country equivalence (Schmitt & Allik, 2005). In our sample, the RSES has a Cronbach's alpha of 0.59 in Ethiopia and 0.51 in Bangladesh. Both measures have been shown to work well among adolescents (see, for example, Stark et al., 2018; and; Tait, French, & Hulse, 2003). Summary statistics of these variables can also be found in Table 1b, Table 1aa and 1b.

### 2.4 Analysis

We utilize regression analysis to explore the association between gender attitudes and norms of the adolescent on their health outcomes. We utilize ordinary least squares for both continuous and binary (linear probability model [LPM]) outcomes. We show results from the LPM instead of a logistic regression for ease of discussion and comparability of results across outcomes (results are robust to the use of logistic regression). All models utilize sample weights to make the results representative of the study areas and cluster the standard errors at the community or school level. We also control for the set of variables used in sampling. These include an indicator variable for whether the adolescent is female, location indicators, an indicator for whether there were multiple eligible adolescents in the household, and an indicator for whether the adolescent was part of the purposely sampled subset of the data.

In addition, although not the focus of this paper, to more directly follow the framework of Cislaghi and Heise (2018), we also control for measures of material and social factors in our analysis. We create asset quintiles using principal component analysis (Filmer & Pritchett, 2001) to capture material factors and include an indicator for having friends that the adolescent trusts, as well as an index of whether or not s/he talks to his/her female caregiver about work, education, bullying, or religion (0–4). Because nonresponse is relatively low, we restrict our analysis to the subset of adolescents that answered all questions.

For each outcome of interest, we first explore relationships within the full sample before investigating heterogeneity by gender and location. We include in the regression a standardized index of individual attitudes and a community-level standardized index of norms (see Clark et al., 2018 for a similar approach). Examining attitudes at the individual level and norms at the community level mirrors the framework of Cislaghi and Heise (2018).

## 3 Results

Table 1b, Table 1aa and 1b displays the summary statistics for the main independent and dependent variables of interest for Bangladesh and Ethiopia, respectively. Throughout this analysis, when we refer to attitudes and norms being more restrictive or more gendered, we mean that the respondent adheres to gender stereotypes related to males or females. RGAs and RGNs are pervasive in both countries, with Bangladeshi adolescents scoring an average of 5.62 out of 16 on attitudes and 7.34 out of 14 on norms, and Ethiopian adolescents scoring an average of 6.59 and 9.47, respectively. These numbers show that norms are sharply more gendered than attitudes. There is considerable variation in both countries. In Bangladesh, attitudes vary from 5.35 (boys) to 5.96 (rural) and norms vary from 7.33 (urban) to 7.40 (rural). In Ethiopia, attitudes vary from 5.56 (urban) to 6.68 (girls) and norms vary from 7.73 (urban) to 9.66 (rural)—note the higher RGNs in rural Ethiopia than in rural Bangladesh. These total scores mask substantial heterogeneity in the underlying attitudes and norms (see Appendix Tables A1–A4) that deserves future investigation. In both countries, questions that were worded so that agreement indicated a nongendered response appear to elicit less restrictive gendered attitudes and norms than the reverse, which also merits additional research.

Table 2 through 5 have the same structure. Columns 1 through 5 show results for Bangladesh and columns 6 through 10 show results for Ethiopia. Columns 1 and 6 display overall results, columns 2 and 7 results for girls, columns 3 and 8 results for boys, columns 4 and 9 results for rural locations, and columns 5 and 10 results for urban locations.

Table 2 reports the results on HAZ and BMIZ. First looking at HAZ in Bangladesh, although the coefficients on RGAs and RGNs are both negative in column 1, the coefficients are insignificant. For Ethiopia on the other hand, we see a significant negative relationship between RGAs and HAZ (-0.069; *P* < .05) but no relationship with RGNs. In both Bangladesh and Ethiopia, we see evidence of a negative and significant association of RGAs with HAZ for boys, with relatively similar coefficients (-0.101, *P* < .1 in Bangladesh; -0.104, *P* < .01 in Ethiopia); we find no evidence of a relationship for girls. There is limited evidence of heterogeneity by urban or rural location. In terms of BMIZ, we see no association with RGAs and RGNs in Bangladesh overall or for any subgroup (Table 2, Panel B, columns 1–5). In Ethiopia, coefficients on RGAs are once again negative and significant (-0.031; *P* < .05), with similar coefficients for boys and girls. The association appears stronger in urban areas, where RGNs are also associated with lower BMI (-0.115 *P* < .05).

**Table 2:** Height-for-age and body mass index-for-age Z-scores.

Panel A: Height-for-Age Z-Score										
	Bangladesh					Ethiopia				
	Overall	Girls	Boys	Rural	Urban	Overall	Girls	Boys	Rural	Urban
	(1)	(2)	(3)	(4)	(5)	(6)	(7)	(8)	(9)	(10)
Standardized index of restrictive gender attitudes (RGAs)	-0.013	0.075	-0.101*	0.006	-0.020	-0.069***	-0.030	-0.104***	-0.069**	-0.067
	(0.046)	(0.060)	(0.058)	(0.074)	(0.054)	(0.025)	(0.029)	(0.035)	(0.027)	(0.043)
Standardized index of restrictive gender norms (RGNs)	-0.097	-0.094	-0.089	-0.075	-0.082	0.023	-0.007	0.049	0.023	0.047
	(0.065)	(0.086)	(0.086)	(0.159)	(0.069)	(0.037)	(0.039)	(0.051)	(0.042)	(0.051)
Sample size	1,620	839	781	451	1,169	4,759	2,651	2,108	4,034	725
Adjusted R2	0.100	0.097	0.095	0.056	0.122	0.135	0.127	0.128	0.131	0.121
Panel B: BMI-for-Age Z-Score										
	Bangladesh					Ethiopia				
	Overall	Girls	Boys	Rural	Urban	Overall	Girls	Boys	Rural	Urban
	(1)	(2)	(3)	(4)	(5)	(6)	(7)	(8)	(9)	(10)
Standardized index of RGAs	-0.063	-0.028	-0.079	-0.077	-0.057	-0.031**	-0.033*	-0.035	-0.030*	-0.071
	(0.046)	(0.068)	(0.063)	(0.055)	(0.054)	(0.016)	(0.019)	(0.024)	(0.016)	(0.048)
Standardized index of RGNs	0.003	-0.101	0.089	0.010	0.015	0.010	0.010	0.008	0.035	-0.115**
	(0.073)	(0.087)	(0.107)	(0.114)	(0.083)	(0.025)	(0.033)	(0.033)	(0.027)	(0.046)
Sample size	1,620	839	781	451	1,169	4,759	2,651	2,108	4,034	725
Adjusted R2	0.090	0.077	0.109	0.039	0.089	0.092	0.109	0.083	0.064	0.050

Notes: Regressions are ordinary least squares (OLS) models, with coefficients. All regressions are weighted to make them representative of the target population in the study communities and standard errors are clustered at the community level. The following variables are included as controls in the regression analyses: asset deciles, indicator for having friends that he/she trusts, an index of whether or not he/she talks to her female caregiver about work, education, bulling, or religion (0–4), an indicator variable for whether the adolescent is female, location indicators, an indicator for whether the household had multiple eligible adolescents, and an indicator for whether the adolescent was part of the purposely sampled subset of the data. Parameter estimates statistically different than zero at 99% (***), 95% (**), and 90% (*) confidence.

Table 3 presents the evidence on self-reported health and illness. For Bangladesh, we find a positive and significant association between RGAs and self-reported health (Table 3, Panel A)—a 1 SD increase in RGAs is associated with a 2.8 percentage point (pp) increase in self-reported health (*P* < .05), with no impact on illness (Table 3, Panel B). The positive association with health appears largely driven by boys (0.036; *P* < .05). In Ethiopia, a 1 SD increase in RGAs is associated with a 1.5 pp decline in self-reported health (*P* < .05), with no impact of RGNs. This association is stronger for boys and in rural areas. We find no overall relationship between RGAs or RGNs and illness in either location, although coefficients are generally negative.

**Table 3:** Self-reported health and illness or injury.

Panel A: = 1 if Self-Reported Health Good or Very Good										
	Bangladesh					Ethiopia				
	Overall	Girls	Boys	Rural	Urban	Overall	Girls	Boys	Rural	Urban
	(1)	(2)	(3)	(4)	(5)	(6)	(7)	(8)	(9)	(10)
Standardized index of restrictive gender attitudes (RGAs)	0.028**	0.020	0.036**	0.025*	0.027	-0.015**	-0.013	-0.016*	-0.017**	-0.002
	(0.014)	(0.023)	(0.016)	(0.014)	(0.017)	(0.006)	(0.008)	(0.008)	(0.006)	(0.013)
Standardized index of restrictive gender norms (RGNs)	0.004	-0.016	0.025	-0.015	0.011	-0.014	-0.009	-0.019	-0.013	-0.021*
	(0.017)	(0.020)	(0.025)	(0.010)	(0.021)	(0.009)	(0.009)	(0.012)	(0.010)	(0.011)
Sample size	1,623	841	782	451	1,172	4,866	2,712	2,154	4,126	740
Adjusted R2	0.007	0.003	0.019	0.049	0.001	0.046	0.045	0.047	0.044	0.064
Panel B: = 1 if Adolescent Experienced Health Symptom in Past 4 Weeks or Serious Illness or Injury in Past 12 Months										
	Bangladesh					Ethiopia				
	Overall	Girls	Boys	Rural	Urban	Overall	Girls	Boys	Rural	Urban
	(1)	(2)	(3)	(4)	(5)	(6)	(7)	(8)	(9)	(10)
Standardized index of RGAs	-0.002	-0.023	0.016	-0.032*	0.003	-0.004	-0.000	-0.007	-0.001	-0.014
	(0.015)	(0.018)	(0.021)	(0.018)	(0.017)	(0.012)	(0.013)	(0.016)	(0.013)	(0.022)
Standardized index of RGNs	-0.008	0.007	-0.027	0.020	-0.013	-0.016	-0.006	-0.024	-0.024	0.037
	(0.016)	(0.019)	(0.023)	(0.034)	(0.017)	(0.016)	(0.019)	(0.019)	(0.018)	(0.038)
Sample size	1,622	841	781	451	1,171	4,860	2,710	2,150	4,120	740
Adjusted R2	0.009	0.003	0.017	-0.001	0.014	0.016	0.030	0.007	0.013	0.010

Notes: Regressions are ordinary least squares (OLS) models, with coefficients. All regressions are weighted to make them representative of the target population in the study communities and standard errors are clustered at the community level. The following variables are included as controls in the regression analyses: asset deciles, indicator for having friends that he/she trusts, an index of whether or not he/she talks to her female caregiver about work, education, bulling, or religion (0–4), an indicator variable for whether the adolescent is female, location indicators, an indicator for whether the household had multiple eligible adolescents, and an indicator for whether the adolescent was part of the purposely sampled subset of the data. Parameter estimates statistically different than zero at 99% (***), 95% (**), and 90% (*) confidence.

Table 4 shows the results on adolescent self-reported hunger. A 1 SD increase in RGAs is associated with a 1.2 pp (*P* < .1) increase in the probability of the adolescent reporting that s/he is hungry, with a stronger association in urban areas. We see no association between RGNs and hunger. In Ethiopia, in contrast, we so no relationship between RGAs and hunger but evidence of a strong relationship between RGNs and hunger. A 1 SD increase in RGNs is associated with a 5.5 pp (*P* < .01) increase in the likelihood that the adolescent experiences hunger, with a stronger association for boys.

**Table 4:** Adolescent hunger.

Panel A: = 1 if Adolescent Experienced Hunger in the Past Four Weeks										
	Bangladesh					Ethiopia				
	Overall	Girls	Boys	Rural	Urban	Overall	Girls	Boys	Rural	Urban
	(1)	(2)	(3)	(4)	(5)	(6)	(7)	(8)	(9)	(10)
Standardized index of restrictive gender attitudes (RGAs)	0.012*	0.015*	0.011	0.004	0.014**	-0.002	0.014	-0.018	-0.003	0.001
	(0.006)	(0.009)	(0.010)	(0.023)	(0.006)	(0.009)	(0.010)	(0.013)	(0.010)	(0.014)
Standardized index of restrictive gender norms (RGNs)	-0.001	-0.007	0.006	0.005	-0.002	0.055***	0.024*	0.086***	0.058***	0.034**
	(0.004)	(0.005)	(0.006)	(0.012)	(0.004)	(0.013)	(0.013)	(0.017)	(0.015)	(0.015)
Sample size	1,623	841	782	451	1,172	4,865	2,711	2,154	4,126	739
Adjusted R2	0.052	0.048	0.050	0.027	0.057	0.072	0.057	0.083	0.070	0.083

Notes: Regressions are ordinary least squares (OLS) models, with coefficients. All regressions are weighted to make them representative of the target population in the study communities and standard errors are clustered at the community level. The following variables are included as controls in the regression analyses: asset deciles, indicator for having friends that he/she trusts, an index of whether or not he/she talks to her female caregiver about work, education, bulling, or religion (0–4), an indicator variable for whether the adolescent is female, location indicators, an indicator for whether the household had multiple eligible adolescents, and an indicator for whether the adolescent was part of the purposely sampled subset of the data. Parameter estimates statistically different than zero at 99% (***), 95% (**), and 90% (*) confidence.

Table 5 presents results from the GHQ-12 and the RSES. In Bangladesh, a 1 SD increase in RGAs is associated with an increase (worsening) in the GHQ-12 by 0.165 (*P* < .01). These results are stronger for girls and in urban areas. On the other hand, a 1 SD increase in RGNs has a significant and negative association (improvement) with the GHQ-12 (-0.200; *P* < 0.01). This negative association is driven by substantial urban or rural heterogeneity, with the sign flipping to positive in rural areas—adolescents in rural areas see an increase (worsening) in the GHQ-12 with increased RGNs. In Ethiopia, both RGAs (0.149; *P* < .01) and RGNs (0.207; *P* < .01) are strongly positively associated with the GHQ-12. The relationship between RGAs and the RSES is significant and large in both countries—a 1 SD increase in RGAs decreases (worsens) the RSES by 0.744 (*P* < .01) in Bangladesh and by 0.687 (*P* < .01) in Ethiopia. There is no overall impact of RGNs. The relationship is exacerbated for girls and in urban areas in both countries.

**Table 5:** General health questionnaire 12 and Rosenberg's self-esteem scale.

Panel A: General Health Questionnaire 12 Index (0–12; Higher Indicates Worse Mental Health)										
	Bangladesh					Ethiopia				
	Overall	Girls	Boys	Rural	Urban	Overall	Girls	Boys	Rural	Urban
	(1)	(2)	(3)	(4)	(5)	(6)	(7)	(8)	(9)	(10)
Standardized index of restrictive gender attitudes (RGAs)	0.165***	0.188***	0.160	0.008	0.218***	0.149***	0.142***	0.155***	0.154***	0.107*
	(0.055)	(0.061)	(0.099)	(0.072)	(0.068)	(0.037)	(0.044)	(0.049)	(0.041)	(0.062)
Standardized index of restrictive gender norms (RGNs)	-0.200***	-0.217**	-0.195**	0.227*	-0.316***	0.207***	0.196***	0.213***	0.205***	0.204***
	(0.073)	(0.087)	(0.096)	(0.120)	(0.073)	(0.044)	(0.056)	(0.055)	(0.050)	(0.065)
Sample size	1,613	834	779	451	1,162	4,857	2,706	2,151	4,119	738
Adjusted R2	0.041	0.046	0.033	0.061	0.051	0.040	0.037	0.046	0.042	0.031
Panel B: Rosenberg's Self-Esteem Scale (10–40; Larger Values Equal Higher Self-esteem)										
	Bangladesh					Ethiopia				
	Overall	Girls	Boys	Rural	Urban	Overall	Girls	Boys	Rural	Urban
	(1)	(2)	(3)	(4)	(5)	(6)	(7)	(8)	(9)	(10)
Standardized index of RGAs	-0.744***	-0.955***	-0.603***	-0.469	-0.826***	-0.687***	-0.719***	-0.651***	-0.658***	-1.022***
	(0.126)	(0.170)	(0.194)	(0.324)	(0.128)	(0.074)	(0.084)	(0.091)	(0.080)	(0.155)
Standardized index of RGNs	-0.093	0.117	-0.262	-0.143	-0.091	0.132	0.287**	-0.008	0.183	-0.200
	(0.172)	(0.285)	(0.225)	(0.268)	(0.200)	(0.120)	(0.126)	(0.150)	(0.136)	(0.177)
Sample size	1,610	833	777	450	1,160	4,856	2,706	2,150	4,117	739
Adjusted R2	0.068	0.072	0.070	0.044	0.079	0.076	0.077	0.077	0.067	0.119

*Notes:* Regressions are ordinary least squares (OLS) models, with coefficients. All regressions are weighted to make them representative of the target population in the study communities and standard errors are clustered at the community level. The following variables are included as controls in the regression analyses: asset deciles, indicator for having friends that he/she trusts, an index of whether or not he/she talks to her female caregiver about work, education, bulling, or religion (0–4), an indicator variable for whether the adolescent is female, location indicators, an indicator for whether the household had multiple eligible adolescents, and an indicator for whether the adolescent was part of the purposely sampled subset of the data. Parameter estimates statistically different than zero at 99% (***), 95% (**), and 90% (*) confidence.

## 4 Discussion

The results suggest an important but nuanced role for RGAs and RGNs as drivers of physical and mental health among young adolescents (10–12) in Bangladesh and Ethiopia. In particular, these results highlight the role of gender norms in influencing adolescent capabilities and reflect increasing evidence on the multifaceted vulnerabilities of adolescents (Blum et al., 2017; GAGE Consortium, 2017; Harper et al., 2018). They also point to the fact that girls and boys are both vulnerable to restrictive gender attitudes and norms and illustrate the complicated relationship between individual attitudes and collective community norms.

In terms of *physical health*, the negative associations between RGAs and HAZ in Ethiopia (significant) and Bangladesh (insignificant) are particularly concerning given the strong evidence of the potential for sustained negative impacts into adulthood (Hoddinott et al., 2013) and for intergenerational impacts (Patton et al., 2018). Interestingly, this negative association is strongest among boys in both countries. We also see a negative association between RGAs and BMIZ in both Bangladesh (insignificant) and Ethiopia (significant). These stronger associations in Ethiopia may be driven by the significantly poorer health status of the Ethiopian sample because of poor nutrition in childhood (average HAZ is -0.780 in Ethiopia vs. 0.331 in Bangladesh) as well as current nutrition (average BMIZ is -1.151 in Ethiopia vs. -0.055 in Bangladesh). These results also align with the negative association of RGAs and self-reported health in Ethiopia.

Furthermore, the significant and positive relationship of RGAs in Bangladesh and RGNs in Ethiopia with adolescents' self-reports of experiencing hunger shows the importance of exploring relationships at the individual level within the household regarding unequal intra-household power relations, including allocation of resources such as food (Briones, Cockx, & Swinnen, 2018), and may indicate that adolescents' nutritional needs are different from adults’ needs (Das et al., 2017). In Ethiopia, this result is also consistent with the BMIZ result and the self-reported health results discussed above.

Turning to *mental health*, we find a strong and large positive association between RGAs and the GHQ-12 (indicating worse mental health) in both Bangladesh and Ethiopia. This impact exists across gender and rural/urban location, and the overall effect is consistent across countries (0.165 in Bangladesh and 0.149 in Ethiopia). However, we find a divergent association between RGNs and the GHQ-12—with a positive association in Ethiopia but a negative association in Bangladesh. The negative association between the GHQ-12 and RGNs in Bangladesh hides substantial heterogeneity by location, with a strong negative association driven by urban adolescents and a positive association that aligns with Ethiopia in rural locations. In Ethiopia, we also see that the RGNs remain important predictors of mental health in urban areas. This divergence in results between urban Bangladesh and urban Ethiopia may reflect the more restrictive gender norms in urban Ethiopia than in Dhaka, where girls have greater relative independence partially because of increased access to decent paid work. Moreover, in Bangladesh, urban communities are more established, while in Ethiopia the majority of our urban sample are first generation, which may present a unique set of challenges.

We also see a very consistent story across the two countries with regard to self-esteem, with a strong negative association between RGAs and the RSES in both contexts. This result holds for all subgroups, with suggestive evidence on larger impacts for girls and in urban areas. This stronger association for girls may reflect the fact that girl's self-esteem is strongly linked with social isolation and mobility restriction. Moreover, because of higher perceived violence in urban areas, the impact is stronger in urban areas.

Although we have highlighted some important differences in how RGNs and RGAs impact health by urban or rural location and across countries, we find limited evidence of significant gender differences within country on any of the health outcomes. This shows that RGAs and RGNs affect both boys and girls, reflecting increasing evidence that gender inequality is undesirable for everyone (Blum et al., 2017; Mmari et al., 2018). This result is important because it suggests that focusing gender programs solely on adolescent girls is misguided when tackling gender discrimination.

### 4.1 Limitations

There are some important limitations in our analysis. First, these results reflect correlation rather than causation and should be interpreted in this light. Second, the Cronbach's alpha on the attitude measure is lower than we would like, and this measure needs further refinement. Third, although we think it is unlikely, we cannot rule out that differences in social desirability bias in self-reported attitudes and norms between countries or across groups drive some of the results. Similarly, questions about norms are often conceptually challenging for young people. Fourth, comparing outcomes between older (15–17) and younger (10–12) adolescents would enhance our understanding of how the impacts of RGAs and RGNs manifest throughout adolescence. Unfortunately, we cannot make that comparison here as we only interviewed older adolescents in urban areas, which would make it impossible to determine whether any differences between older and younger adolescents is due to the urban location or the adolescent getting older. Finally, the current work examines the full set of attitude and norm questions, likely masking important heterogeneity across different domains.

### 4.2 Conclusions

Our results highlight the powerful role that distal factors such as culture and beliefs, as manifested through restrictive gender attitudes and norms, can play in shaping health outcomes, particularly mental health. In addition, our findings support the importance of multisectoral, age-appropriate investments during the first 8,000 days from conception, as proposed by Bundy, de Silva, Horton, Jamison, and Patton (2017). In particular, it is worth highlighting three key findings from our analysis. First, RGAs and RGNs negatively impact the health of both boys and girls in these two diverse contexts, suggesting that programming should not focus solely on one gender. Second, the increase in rural-to-urban migration in many LMICs appears to be leaving urban youth particularly vulnerable, suggesting that this is a population that might need targeted interventions. Finally, the consistent negative, large, and highly significant association between RGAs and self-esteem in both countries is worth further investigation.

Ultimately, more research that unpacks these key findings as well as more broadly attempts to understand the underlying drivers of restrictive gender attitudes and norms will be essential in improving adolescents’ lives. Specifically, a clearer understanding of the role of individual attitudes versus community norms (and measurement of both) and how they interact and influence each other is vital for better programming. Specifically, unpacking the impact of having individual attitudes that deviate from community norms is an avenue for future research. Moreover, additional inquiry into some of the urban or rural and cross-country differences could shed light on how to design future programs and policies. In summary, working with communities and developing programming to reshape these attitudes and norms, in combination with tackling the other drivers of health such as wealth through a multisectoral approach, is likely not only to improve the current well-being of adolescents but also to reap subsequent dividends in improved outcomes throughout the life course.

## Funding sources

GAGE is a longitudinal research program funded by the Research and Evidence Division of the UK Department for International Development as part of UK aid.

## Ethical approval

The GAGE research program was approved by the George Washington University Committee on Human Research, Institutional Review Board (071721), the ODI Research Ethics Committee (02438), the Ethiopian Development Research Institute (EDRI/DP/00689/10), the Addis Ababa University College of Health Sciences Institutional Review Board (113/17/Ext), and the Human Subjects Committee for Innovations for Poverty Action IRB-USA (14160).

## Declaration of competing interest

None.

## Figures and Tables

**Table A.1:** Measures of Restrictive Gender Attitudes (Bangladesh)

	Overall	Male	Female	Rural	Urban
Educationrowhead					
If a family can afford for one child to go to secondary school, it should be the boy only	0.230	0.167	0.295	0.256	0.223
Only boys should learn about science, technology, and math	0.117	0.084	0.150	0.155	0.105
Girls should be sent to school only if they are not needed to help at home	0.174	0.167	0.181	0.159	0.178
Girls should avoid raising their voice to be ladylike	0.745	0.764	0.725	0.712	0.754
(Reverse) Boys should be able to show their feelings without fear of being teased	0.714	0.766	0.662	0.738	0.707
Time Userowhead					
(Reverse) Girls and boys should share household tasks equally	0.137	0.127	0.148	0.119	0.143
A woman's most important role is to take care of her home and cook for her family	0.570	0.533	0.609	0.685	0.537
A man should have the final word on decisions in his home	0.522	0.473	0.573	0.512	0.525
Financial Inclusion and Economic Empowermentrowhead					
(Reverse) Women should have the same chance to work outside of the home as men	0.111	0.0847	0.139	0.193	0.087
Women who participate in politics or leadership positions cannot also be good wives or mothers	0.408	0.366	0.451	0.468	0.390
(Reverse) It is important for women and adolescent girls to have their own savings	0.107	0.0656	0.150	0.144	0.097
Relationships and Marriagerowhead					
(Reverse) A boy should be able to have a girlfriend if he wants to	0.174	0.160	0.189	0.167	0.176
(Reverse) A girl should be able to have a boyfriend if she wants to	0.186	0.181	0.190	0.181	0.187
(Reverse) A girl's marriage can wait until she has completed secondary school	0.208	0.182	0.235	0.243	0.198
Sexual and Reproductive Healthrowhead					
(Reverse) Girls should be proud of their bodies as they become women	0.461	0.463	0.459	0.467	0.460
Families should control their daughters' behaviors more than their sons'	0.754	0.767	0.740	0.755	0.753
Sample size	1623	841	782	451	1172

*Notes:* This table summarizes the gender attitudes from the Gender and Adolescence: Global Evidence (GAGE) Bangladesh quantitative survey. All variables are constructed to take a value of 1 if the response is gendered and 0 otherwise. Means are weighted to make them representative of the study communities.

**Table A.2:** Measures of Restrictive Gender Attitudes (Ethiopia)

	Overall	Male	Female	Rural	Urban
Educationrowhead					
If a family can afford for one child to go to secondary school, it should be the boy only	0.391	0.337	0.439	0.409	0.226
Only boys should learn about science, technology, and math	0.252	0.227	0.275	0.264	0.154
Girls should be sent to school only if they are not needed to help at home	0.323	0.291	0.351	0.335	0.210
Girls should avoid raising their voice to be ladylike	0.633	0.608	0.655	0.642	0.552
(Reverse) Boys should be able to show their feelings without fear of being teased	0.216	0.236	0.198	0.216	0.214
Time Userowhead					
(Reverse) Girls and boys should share household tasks equally	0.364	0.359	0.368	0.381	0.214
A woman's most important role is to take care of her home and cook for her family	0.828	0.823	0.832	0.846	0.662
A man should have the final word on decisions in his home	0.795	0.773	0.814	0.811	0.650
Financial Inclusion and Economic Empowermentrowhead					
(Reverse) Women should have the same chance to work outside of the home as men	0.189	0.181	0.197	0.196	0.133
Women who participate in politics or leadership positions cannot also be good wives or mothers	0.461	0.454	0.467	0.474	0.343
(Reverse) It is important for women and adolescent girls to have their own savings	0.116	0.112	0.119	0.120	0.0823
Relationships and Marriagerowhead					
(Reverse) A boy should be able to have a girlfriend if he wants to	0.361	0.428	0.301	0.357	0.403
(Reverse) A girl should be able to have a boyfriend if she wants to	0.426	0.480	0.376	0.425	0.429
(Reverse) A girl's marriage can wait until she has completed secondary school	0.224	0.204	0.243	0.220	0.263
Sexual and Reproductive Healthrowhead					
(Reverse) Girls should be proud of their bodies as they become women	0.215	0.193	0.234	0.210	0.252
Families should control their daughters' behaviors more than their sons'	0.798	0.783	0.812	0.801	0.776
Sample size	4,866	2,712	2,154	4,126	740

*Notes:* This table summarizes the gender attitudes from the Gender and Adolescence: Global Evidence (GAGE) Ethiopia quantitative survey. All variables are constructed to take a value of 1 if the response is gendered and 0 otherwise. Means are weighted to make them representative of the study communities.

**Table A.3:** Measures of Community-Level Restrictive Gender Norms (Bangladesh)

	Overall	Rural	Urban
Educationrowhead			
Our culture makes it harder for girls to achieve their goals than boys	0.693	0.678	0.697
Adolescent girls in my community are more likely to be out of school than adolescent boys	0.376	0.322	0.391
Girls in my community are sent to school only if they are not needed to help at home	0.210	0.246	0.200
Most people in my community expect girls to be sent to school only if they are not needed at home	0.232	0.252	0.227
Time Userowhead			
Most boys and girls in my community do not share household tasks equally	0.715	0.766	0.701
Most people in my community expect men to have the final word about decisions in the home	0.551	0.569	0.546
Most people in my community do not expect girls and boys to share household tasks equally	0.747	0.755	0.745
Most men in my community are the ones who make the decisions in their home	0.662	0.638	0.668
Financial Inclusion and Economic Empowermentrowhead			
(Reverse) Most women in my community have the same chance to work outside the home as men	0.343	0.409	0.324
(Reverse) Most people in my community expect women to have the same chance to work outside the home as men	0.343	0.391	0.329
Relationships and Marriagerowhead			
Most adolescent girls in my community marry before the age of 18 years	0.485	0.435	0.499
Adults in my community expect adolescent girls to get married before the age of 18 years	0.330	0.374	0.318
Sexual and Reproductive Healthrowhead			
Most families in my community control their daughters' behaviors more than their sons'	0.812	0.814	0.811
Most people in my community expect families to control their daughter's behavior more than their sons'	0.824	0.803	0.830
Sample size: Community	111	36	75

*Notes:* This table summarizes the gender norms from the Gender and Adolescence: Global Evidence (GAGE) Bangladesh quantitative survey. All variables are constructed to take a value of 1 if the response is gendered and 0 otherwise. The mean of these variables is then constructed at the community level. Means are weighted to make them representative of the study communities.

**Table A.4:** Measures of Community-Level Restrictive Gender Norms (Ethiopia)

	Overall	Rural	Urban
Educationrowhead			
Our culture makes it harder for girls to achieve their goals than boys	0.664	0.685	0.503
Adolescent girls in my community are more likely to be out of school than adolescent boys	0.704	0.714	0.626
Girls in my community are sent to school only if they are not needed to help at home	0.427	0.440	0.318
Most people in my community expect girls to be sent to school only if they are not needed at home	0.504	0.516	0.415
Time Userowhead			
Most boys and girls in my community do not share household tasks equally	0.805	0.817	0.706
Most people in my community expect men to have the final word about decisions in the home	0.865	0.884	0.712
Most people in my community do not expect girls and boys to share household tasks equally	0.862	0.874	0.769
Most men in my community are the ones who make the decisions in their home	0.864	0.875	0.776
Financial Inclusion and Economic Empowermentrowhead			
(Reverse) Most women in my community have the same chance to work outside the home as men	0.397	0.413	0.275
(Reverse) Most people in my community expect women to have the same chance to work outside the home as men	0.427	0.436	0.362
Relationships and Marriagerowhead			
Most adolescent girls in my community marry before the age of 18 years	0.626	0.662	0.341
Adults in my community expect adolescent girls to get married before the age of 18 years	0.595	0.630	0.318
Sexual and Reproductive Healthrowhead			
Most families in my community control their daughters' behaviors more than their sons'	0.867	0.867	0.864
Most people in my community expect families to control their daughter's behavior more than their sons'	0.866	0.866	0.864
Sample size: Community	218	175	43

*Notes:* This table summarizes the gender norms from the Gender and Adolescence: Global Evidence (GAGE) Ethiopia quantitative survey. All variables are constructed to take a value of 1 if the response is gendered and 0 otherwise. The mean of these variables is then constructed at the community level. Means are weighted to make them representative of the study communities.

**Table A.5:** Source of Measures of Restrictive Gender Attitudes and Norms

	Attitude or Norm	Scale (Verbatim or Adapted)	Source
Educationrowhead			
If a family can afford for one child to go to secondary school, it should be the boy only	Attitude	Gender Attitudes Scale	Lundgren et al. (2013)
Only boys should learn about science, technology, and math	Attitude	Gender and Adolescence: Global Evidence	New
Girls should be sent to school only if they are not needed to help at home	Attitude	Gender Attitudes Scale	Lundgren et al. (2013)
Girls should avoid raising their voice to be ladylike	Attitude	Gender Stereotypical Traits	Global Early Adolescent Study (2018)
(Reverse) Boys should be able to show their feelings without fear of being teased	Attitude	Gender Stereotypical Traits	Global Early Adolescent Study (2018)
Our culture makes it harder for girls to achieve their goals than boys	Norm	Gender and Adolescence: Global Evidence	New
Adolescent girls in my community are more likely to be out of school than adolescent boys	Norm	Gender and Adolescence: Global Evidence	New
Girls in my community are sent to school only if they are not needed to help at home	Norm	Gender Attitudes Scale	Lundgren et al. (2013)
Most people in my community expect girls to be sent to school only if they are not needed at home	Norm	Gender Attitudes Scale	Lundgren et al. (2013)
Time Userowhead			
(Reverse) Girls and boys should share household tasks equally	Attitude	Gender Stereotypical Roles	Global Early Adolescent Study (2018)
A woman's most important role is to take care of her home and cook for her family	Attitude	Gender Equitable Men (GEM) Scale	Vu et al. (2017)
A man should have the final word on decisions in his home	Attitude	GEM Scale	Vu et al. (2017)
Most boys and girls in my community do not share household tasks equally	Norm	Gender Stereotypical Roles	Global Early Adolescent Study (2018)
Most people in my community expect men to have the final word about decisions in the home	Norm	GEM Scale	Vu et al. (2017)
Most people in my community do not expect girls and boys to share household tasks equally	Norm	Gender Stereotypical Roles	Global Early Adolescent Study (2018)
Most men in my community are the ones who make the decisions in their home	Norm	International Men and Gender Equality Survey (IMAGES)	Levtov et al. (2018)
Financial Inclusion and Economic Empowermentrowhead			
(Reverse) Women should have the same chance to work outside of the home as men	Attitude	Gender Attitudes Scale	Lundgren et al. (2013)
Women who participate in politics or leadership positions cannot also be good wives or mothers	Attitude	IMAGES	Levtov et al. (2018)
(Reverse) It is important for women and adolescent girls to have their own savings	Attitude	Gender and Adolescence: Global Evidence	New
(Reverse) Most women in my community have the same chance to work outside the home as men	Norm	Gender Attitudes Scale	Lundgren et al. (2013)
(Reverse) Most people in my community expect women to have the same chance to work outside the home as men	Norm	Gender Attitudes Scale	Lundgren et al. (2013)
Relationships and Marriagerowhead			
(Reverse) A boy should be able to have a girlfriend if he wants to	Attitude	Adolescent Relationship Expectations	Global Early Adolescent Study (2018)
(Reverse) A girl should be able to have a boyfriend if she wants to	Attitude	Adolescent Relationship Expectations	Global Early Adolescent Study (2018)
(Reverse) A girl's marriage can wait until she has completed secondary school	Attitude	Schooling, Income and Health Risk	Baird et al. (2017)
Most adolescent girls in my community marry before the age of 18 years	Norm	Gender and Adolescence: Global Evidence	New
Adults in my community expect adolescent girls to get married before the age of 18 years	Norm	Gender and Adolescence: Global Evidence	New
Sexual and Reproductive Healthrowhead			
(Reverse) Girls should be proud of their bodies as they become women	Attitude	Gender and Adolescence: Global Evidence	New
Families should control their daughters' behaviors more than their sons'	Attitude	IMAGES	Levtov et al. (2018)
Most families in my community control their daughters' behaviors more than their sons'	Norm	IMAGES	Levtov et al. (2018)
Most people in my community expect families to control their daughter's behavior more than their sons'	Norm	IMAGES	Levtov et al. (2018)

*Notes:* This table provides a source for all the attitude and norm questions in the quantitative survey. Many of these questions are used in multiple sources. All questions that are indicated as developed as part of the Gender and Adolescence: Global Evidence (GAGE) program use concepts from multiple sources.
